# Barriers in access to healthcare for women with disabilities: a systematic review in qualitative studies

**DOI:** 10.1186/s12905-021-01189-5

**Published:** 2021-01-30

**Authors:** Behzad Karami Matin, Heather J. Williamson, Ali Kazemi Karyani, Satar Rezaei, Moslem Soofi, Shahin Soltani

**Affiliations:** 1 Research Center for Environmental Determinants of Health (RCEDH), Health Institute, Kermanshah University of Medical Sciences, Kermanshah, Iran; 2 Northern Arizona University, Flagstaff, USA; 3 Social Development and Health Promotion Research Center, Health Institute, Kermanshah University of Medical Sciences, Kermanshah, Iran

**Keywords:** Systematic review, Intellectual disability, Healthcare delivery, Healthcare disparities, Qualitative research

## Abstract

**Background:**

Studies show that different socio-economic and structural factors can limit access to healthcare for women with disabilities. The aim of the current study was to review barriers in access to healthcare services for women with disabilities (WWD) internationally.

**Methods:**

We conducted a systematic review of relevant qualitative articles in PubMed, Web of Science and Scopus databases from January 2009 to December 2017. The search strategy was based on two main topics: (1) access to healthcare; and (2) disability. In this review, women (older than 18) with different kinds of disabilities (physical, sensory and intellectual disabilities) were included. Studies were excluded if they were not peer-reviewed, and had a focus on men with disabilities.

**Results:**

Twenty four articles met the inclusion criteria for the final review. In each study, participants noted various barriers to accessing healthcare. Findings revealed that WWD faced different sociocultural (erroneous assumptions, negative attitudes, being ignored, being judged, violence, abuse, insult, impoliteness, and low health literacy), financial (poverty, unemployment, high transportation costs) and structural (lack of insurance coverage, inaccessible equipment and transportation facilities, lack of knowledge, lack of information, lack of transparency, and communicative problems) factors which impacted their access healthcare.

**Conclusions:**

Healthcare systems need to train the healthcare workforce to respect WWD, pay attention to their preferences and choices, provide non-discriminatory and respectful treatment, and address stigmatizing attitudinal towards WWD. In addition, families and communities need to participate in advocacy efforts to promote WWD’s access to health care.

## Background

The World Report on Disability in 2011 notes that about 15% (around a billion people) of world population are living with some form of disability [1]. The World Health Survey estimates that the prevalence of disability among women is 60% higher than men [1]. Also, we see a higher rates of disability status in low income countries. In these countries, studies report a higher disability rate among women compared to men [2, 3]. In addition, the literature on healthcare shows that people with disabilities (PWD) experience worse health outcomes compared to their counterparts without disabilities. Among PWD, women with disabilities (WWD) are more likely to have unmet healthcare needs than women without disabilities.

WWD also face different rates of risky health behaviors that affect their health status. Studies indicate that women with intellectual disabilities (WWID) are more likely to report low levels of physical activity and to be overweight compared to women without disabilities [4–6]. Also, some studies indicate that WWD experience greater oral health problems, including a higher prevalence and the greater severity of periodontal diseases than women without disabilities [7–10]^.^ Clearly, there is a necessity to formulate and implement effective policies to improve access to healthcare for WWD. Multiple determinants (e.g. low income, poor education, low-quality health care, etc.) can lead to poorer health status and insufficient access to healthcare for WWD, which in turn impacts their social inclusion [11–13]^.^ Thus support systems need to draw their attention to improve infrastructure and to facilitate access to healthcare as a critical step toward social inclusion of WWD [14].

In past decades, various studies have been completed investigating barriers in access to healthcare for WWD. In the field of sexual and reproductive health (SRH) services, research shows that PWD face outstanding unmet needs and PWD are more likely to be deprived from sex education programs. Some studies identified that people with intellectual disabilities (PWID) have less informal and formal opportunities to learn about sexual health than their counterparts without disabilities [15–17]. Studies also show that the type of disability can affect access to SRH services for PWD. The findings of McCabe and Taleporos indicated that PWID were less likely to report having enough sexual knowledge than people with physical disabilities and the general population [18].

Additionally, WWD face a verity of inequalities to receiving preventive health services, such as screening for breast and cervical cancer in comparison to their counterparts without disabilities [19, 20]. For example, Armour et al. [21] found that WWD in the United States are less likely to report receiving a Pap test than women without disabilities. WWD, due to communicative challenges, mobility impairments and perceptual problems were not able to use Pap tests effectively [22, 23]. Furthermore, studies regarding oral health found that cognitive impairments, fear of treatment, lack of skilled workforces, communicative problems, and lack of dental care services resulted in poorer access to oral health care [10, 24, 25].

A range of different financial, physical, attitudinal and structural barriers have been cited in past studies [26]. Frier et al. [27] found that income, as a social determinant, has the greatest effect on access to healthcare for PWD. Lipson and Rogers investigated the pregnancy, birth and postpartum experiences of women with physical disabilities (WWPD) in the United States. They found that personal factors (such as personality, resources and attitude) and healthcare system factors (such as providers’ attitude, knowledge, structural and political factors) could affect access to maternity care for WWPD in the United States [28].These barriers can differ from one society to another. Developing countries compared to developed countries, have different socio-economic contexts that affect access to healthcare for WWD in different ways. For example, access to various informational resources, like the internet, is more limited in developing countries than developed countries [29–32].

Although quantitative studies mention that WWD are more likely to experience poorer health compared to their counterparts without disabilities, they do not provide enough details and evidence on the nature and the diversity of obstacles experienced by WWD to use healthcare services. Given the role of women in societies and their rights to equally participate in healthcare systems, we decided to make a deeper exploration of the nature and complexity of the barriers experienced by WWD internationally. Accordingly, this literature review specifically focuses on qualitative studies, which can characterize barriers and facilitators to healthcare access for WWD in broader contexts versus quantitative studies.

To acquire a clear and accurate understanding of different types of obstacles in access to healthcare, we decided to categorize the identified barriers according to Levesque’s et al. model [33]. The novelty of this conceptual framework is that Levesque and colleagues identify these dimensions with relevant abilities from the viewpoint of the patient. The relevant abilities comprise: (1) Ability to perceive; (2) Ability to reach; (3) Ability to seek; (4) Ability to pay; and (5) Ability to engage. This conceptual framework has been applied in various studies to investigate access to healthcare among patients [34–36].

Identifying, gathering and analyzing the findings of studies across the world can provide comprehensive information for policy makers and researchers locally, nationally and internationally. The main research question guiding this project was, what do qualitative studies tell us about the barriers experienced by WWD in access to health services internationally? The research question was designed as an open question because access to healthcare is a multidimensional concept, in which many factors can affect access to healthcare in different ways. Given the rapid and continuous changes in economic conditions, medical technologies, communicative tools, assistive devices across the world, we decided to conduct this review within past 10 years. Also, it is important to note that although various qualitative studies have been conducted to explore barriers to participation, physical activity, employment, education and leisure time, in this review, we only included the studies that had been done exploring barriers to healthcare.

## Methods

### Search strategy

**Fig. 1:**
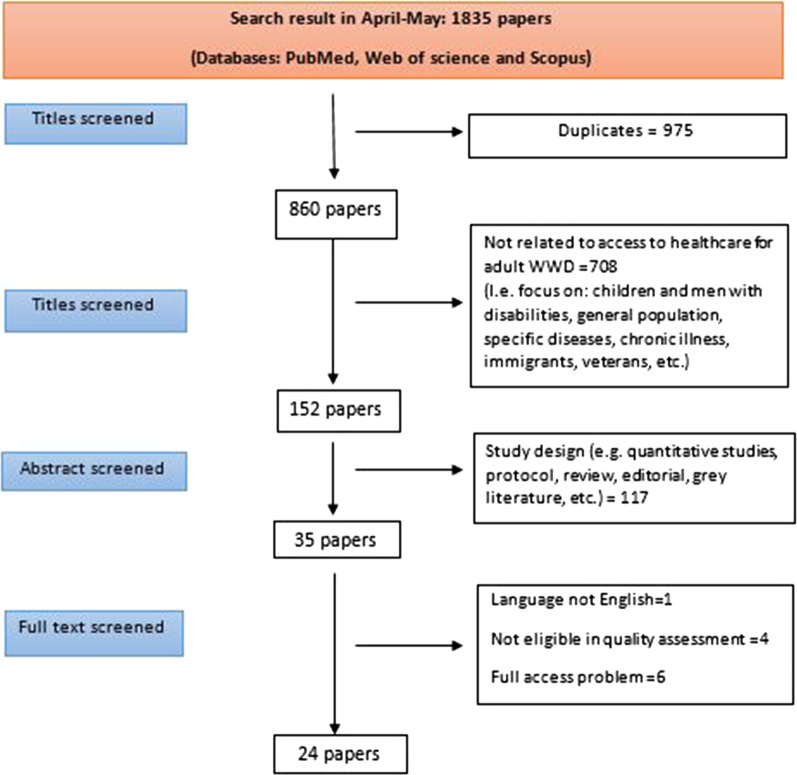
Flowchart of systematic literature search

A structured literature search was done in the bibliographic databases Web of Science, PubMed and Scopus. All papers identified in our searches were exported to EndNote software. The literature search was conducted between April and May 2018. The search strategy was based on two main topics: (1) access to healthcare; and (2) disability. Figure 1 shows the full search strategy used in the study. Also, hand-searching reference lists of research and review papers was used to further identify articles which met our inclusion criteria.

### Selection of studies

According to the aim of study, only qualitative study designs were eligible for inclusion. Thus, observational studies (cross-sectional, prospective and case-control), experimental (randomized controlled and quasi-experimental) and review papers were excluded from the study. In this study, only women (older than 18) with different kinds of physical (e.g. cerebral palsy and spinal cord injury), sensory (e.g. hearing loss) and intellectual disabilities (e.g. Down syndrome) were included. The literature review was limited to articles published between 2009 and 2017. Published papers also needed to be from academic journals and in the English language. The literature review process is shown in Fig. 1.

The process of screening studies was done by one of the authors. First, given the aim of the study, we considered specific criteria to include and exclude studies. Then, an author reviewed the studies following the steps demonstrated in Fig. 1. In case of any difficulty in decisions to exclude or include studies, the author would meet with another author and they would discuss and come to final decision on exclusion or inclusion. It should be noted that our criteria were set before searching studies. The inclusion and exclusion criteria included:

### The inclusion criteria

1. Qualitative studies
2. Women with disabilities (older than 18)
3. Physical, sensory and intellectual disabilities
4. Published in English between 2009 and 2017
5. Studies that were related to access to healthcare
6. Full-text articles

### The exclusion criteria

1. Published before 2009 and after 2017
2. Abstracts, Letter to editor, editorials and comments
3. Method papers or protocols
4. Studies on men and children with disabilities,
5. Grey literature (e.g. conference abstracts, research reports, dissertation, books, policy documents)
6. Non-English language studies
7. Not eligible in quality assessment

### Data extraction

To extract data, we designed a specific form in which information of included articles was gathered according to authors, year, country, sample and perspective, methodology, themes, and main findings. To ensure the validity of gathered information, two members of the study (AK and MS) extracted data from all included studies. Then the corresponding author (SS) checked the accuracy of the data extracted by the authors. In case of any disagreements, we compared all our findings in meetings and resolved them by discussion.

### Quality assessment

It is important to note that because of different methods of data collection (e.g. telephone interviews, focus group and individual interviews) and the role of researchers in interpreting data and reporting findings, there have been continuing debates about quality criteria in qualitative studies in the literature. Some of the proposed questions are whether criteria should be applied at all, which criteria should be used and how to apply them in different studies. The quality criteria for this review are summarized in Table 1. We used the Consolidated Criteria for Reporting Qualitative Research (COREQ) to assess the quality of the qualitative studies [37, 38].

**Table 1:** The study criteria to assess quality of qualitative studies

Topic	Guide question/description
*Title and abstract*	
Title	Does the title of the study describe the nature and topic of the study e.g. qualitative study, healthcare access, phenomenology, women with disabilities, etc.
Abstract	Has the purpose of study, design and approach of the study, participants, the study date and the summary of key findings been provided in the abstract?
*Introduction*	
Context and problem statement	Have description of the problem, its significance, background been explained in the introduction of the study?
Purpose or research question	Have objectives and questions of the study been cited vividly?
*Study design*	
Qualitative approach	What is the methodological orientation of the study? e.g. Grounded theory, content analysis, phenomenology, ethnography
*Participation selection*	
Sampling	How research participants were selected? Purposive, snowball, consecutive, convenience
Description of sample	The needed Details about participants. (E.g. gender, age, kind of disability, marital status, employment status, residence status, etc.)
Sample size	How many participants were in the study?
*Data collection*	
Research team and reflexivity	Has the researcher/interviewer explained about her/his personal characteristics, knowledge, trainings, and experiences in the study?
Method of data collection	How the researcher communicate with the participants? Telephone, individual face to face interview, focus group, etc.
Setting of data collecting	Where was the interview held?
Interview guide	Have the interview questions been provided by the authors in the paper?
Audio/ visual recording	Has the researcher used audio/visual recording to collect the data?
Duration	How long did the interviews last?
*Analysis and findings*	
Description of the coding tree	Has the researcher cited the process of coding qualitative data? e.g. open coding, axial coding and selective coding
Categorization of the study’s findings	Have the study findings been shown in a table? e.g. code, subcategory, category, theme
Data analysis	Has the researcher described the method of data analysis e.g. Thematic, framework, content analysis or grounded theory
Software	Has the researcher used a software to manage the data? e.g. MAXQDA or NVivo

Quality assessment for all included studies was conducted independently by two authors (BKM and SR) using a five-point Likert scale. Each COREQ criteria was scored from 1 to 5 by both researchers and the average score of two researchers was determined as the final score of the quality assessment. We included articles that earned the average score of 3 or higher.

Additionally, Levesque’s et al. model was applied to categorize barriers in access to healthcare among WWD. In this framework, access to healthcare is defined as the opportunity to have health care needs fulfilled. We categorized all barriers into the five dimensions of approachability, acceptability, availability and accommodation, affordability, and appropriateness.

Approachability refers to people’s ability to identify existing healthcare services. Some factors such as transparency can make the services more or less approachable. Acceptability relates to cultural and social aspects that affect access to healthcare like gender, beliefs, education, and race. Availability dimension addresses the issue of whether or not healthcare services are available in the place and at the time that they are needed. Affordability refers to the financial capacity for people to spend resources and time to use appropriate healthcare services. Appropriateness concerns the degree of fit between services and clients needs, its timeliness, the amount of care and the quality of the health services provided [33].

## Results

After the initial search, 1835 records were found. We screened papers according to inclusion and exclusion criteria. At the first step, we studied the title of papers and removed 1683 studies because of duplicates or irrelevant content. At the second step, we studied the abstract of papers and removed 116 papers because of study design (quantitative studies, review, protocol, or editorial). Finally, after studying the full text of the remaining papers, 24 studies met the inclusion criteria.r Table 2 summarizes the overall findings from the 24 included studies according to Levesque’s et al. model.

**Table 2:** General characteristics of included studies

Authors and year	Country	Sample	Types of disabilities	Perspective	Study Design	Study aim	Key issues analyzed	Main findings	Quality score
Barr et al. [55]	United States	N = 42	Physical, sensory and intellectual impairments	WWD	Focus group interviews (n = 6)	To identify barriers to mammography screening among women with different disabilities	Four themes: 1. Access 2. Beliefs 3. Social supports 4. Comfort/accommodations	Barriers: Lack of physical and economic access, skepticism about mammography and vulnerability while receiving care, lack of Sufficient support from people and the facilities, physical discomfort, communication issues, the stress and burden of thinking about arranging for a mammogram	5
Magasi and Hammel [31]	United States	N = 19	Physical impairment	WWD Healthcare providers	Individual interviews and Focus group interviews	To explore women with disabilities’ perceptions of their lived experiences in nursing homes	Five themes: 1. Lost choice, control, and occupational engagement 2. Social isolation 3. Social control 4. The political economy of the nursing home 5. Active resistance	Barriers: Loss of valued occupations and life roles , social isolation, isolation, poverty, lack of affordable and accessible housing, and loss of control, the economic subsequent treatment.motivation in the nursing home industry	4
Lezzoni, Klibridge and Park [79]	United States	N = 20	Physical impairment	WWD	Individual interviews	To explore the perceptions of breast cancer patients with mobility impairments of the physical accessibility of health care equipment and facilities	Three themes: 1. Inaccessible Equipment 2. Access Difficulties 3. Policies and Procedures	Barriers: Inaccessible mammography equipment, examining table, radiation therapy equipment, weight scale, Difficulty positioning while standing and lying down, Inaccessible office doors, equipment are not available when patients come for appointment, Staff injuries while transferring patients,	5
McIlfatrick et al. [42]	UK	N = 18	Intellectual impairment	Healthcare providers	Individual interviews and Focus group interviews	To explore the role of healthcare professionals on supporting women with intellectual disability to access breast screening in one region in the UK	Three themes: 1. Knowledge and awareness of breast cancer and breast screening 2. Role in supporting women with intellectual disability 3. Perceived barriers to women with intellectual disability accessing breast screening services	Barriers: Personal aspects: women’s cognitive deficits, communication and level of understanding, mobility and physical health of women, consent for the procedure and subsequent treatment Lack of carer support, negative carer attitudes, transport and waiting time, healthcare staff’ lack of awareness of intellectual disability	4
Truesdale-Kennedy et al. [46]	UK	N = 19	Intellectual impairment	WWD	Individual semi structured interviews	To understand breast cancer and experiences of breast mammography among women with an intellectual disability	Four themes: 1. Women’s understanding of breast cancer 2. Women’s experiences of breast mammography 3. Perceived barriers to attendance 4. Perceived solutions to barriers	Barriers: Limited knowledge around cancer, the signs and symptoms Lack of information, lack of understanding about the breast screening procedure Feeling of fear, anxiety and embarrassment	5
Gibson and Mykitiuk [80]	Canada	N = 74	Physical, sensory, intellectual, and/or psychiatric impairment	WWD	Focus group interviews (n = 11)	To examine whether the fundamental human rights to physical, social, and psychological health are being upheld in Canada for disabled women	Three themes 1. Labyrinthine health service ‘systems,’ 2. Assumptions, attitudes, and discriminatory practices 3. Inadequate sexual health or reproductive services and supports	Barriers: Disconnected services Lack of communications between of NGOs Lack of coordination between policies and programs Problems to receive financial Assistance and eligibility Erroneous assumptions about capacities and abilities of PWD	5
Morrison et al. [57]	Nepal	N = 27	Physical, sensory, and intellectual impairment	WWD	Semi-structured interviews	To explore disabled women's experiences of maternal and newborn care in rural Nepal	Four themes: 1. Lack of awareness 2. Experience of care 3. Normalcy of pregnancy and home delivery 4. Embarrassment	Barriers: Feeling of shame and embarrassment as a barrier to care seeking and antenatal check-up, lack of awareness about the services available, Being rude, unkind and impolite to WWD, difficulty with communication to WWD, difficulties with the infrastructure and equipment	4
Peters and Cotton [81]	Australia	N = 12	Physical impairment	WWD	Individual semi structured interviews and telephone Interviews	To explore the experiences of breast cancer screening for women with physical disabilities	Four themes: 1. Feeling I’m not in control 2. Being ignored and not listened to 3. Being helpless, alone and afraid 4. Pain, torture and humiliation	Barriers: lack of control and sense of powerlessness, lack of consultation and/or notification, unfamiliar and unfriendly healthcare environment, feeling isolated and ignored, feelings of pain and being tortured	4
Bradbury-Jones et al. [43]	Scotland	N = 5	Physical, sensory, intellectual, and psychiatric impairment	WWD	Individual interviews	To identify how women approach maternity care services, their expectations of services and whether they are able to get the type of care that they need and want	Four themes: 1. Attitudes 2. Knowledge 3. Social norms 4. Perceived control	Barriers: Unfamiliar biomedical jargon, Lack of information or incorrect information, lack of knowledge about health services and their provider, negative past experiences, societal misconceptions about disability and domestic abuse, respecting women’s choices and preferences, lack of involvement in care decisions, fear of disclosure	4
Bradbury-Jones et al. [49]	Scotland	N = 45	NA	Healthcare providers	Focus group interviews (n = 7)	To identify the priority areas for improving access to maternity services for women with disabilities	Two themes: 1. Awareness 2. Disclosure	Barriers: Understandings and awareness of disability and domestic abuse, fear of disclosure among women and professionals	3
Lee et al. [44]	Philippines	N = 32	NA	Healthcare providers	In-depth interviews and Focus group interviews	To examine service providers’ perceptions of disability and their experiences providing sexual and reproductive health services to women with disability.	Five themes: 1. Perceptions of disability, Understanding of the SRH needs and rights of WWD 2. Understanding of violence against WWD 3. Perceptions of barriers to SRH services for WWD 4. Capacity of service providers 5. The role of family	Barriers: Limited awareness, inadequate understanding of women’s rights, little training in relation to disability, limited access to the human and financial resources, negative attitudes, inappropriate behaviors, communication difficulties, stigma, financial dependence, violence or abuse	3
Neille and Penn [51]	South Africa	N = 30 (Female:15, Male:15)	Physical, sensory, psychiatric and intellectual impairment	WWD	Individual semi-structured interviews	To explore barriers to policy implementation and service provision experienced by persons with disabilities living in a rural context	Three themes: 1. Increasing mortality rates 2. Violence 3. Corruption and lack of transparency in government policies and practices	Barriers: Physical Dependence Loss of Friendship Feeling of isolation Difficulties with accessing health, information, education, employment	3
Ganle et al. [45]	Ghana	N = 72	Physical and sensory impairment	WWD	In-depth semi-structured interviews	To explore the challenges women with disabilities encounter in accessing and using institutional maternal healthcare services	Two themes: 1. Desire for children and experiences with pregnancy and childbirth 2. Challenges to maternal healthcare access	Barriers: Mobility problems , limited support, Unfriendly healthcare infrastructure, providers’ insensitivity, Communication problems, lack of knowledge, negative attitudes of service providers	4
Coffey et al. [53]	United States	N = 33 (Female:16 , Male:17)	Physical impairment	WWD	Individual semi-structured interviews	To identify preferred sources of health information and services for persons with physical disability and discover how accessibility could be improved	Four themes: 1. Information sources 2. Medical sources 3. Support groups 4. Access	Barriers: Lack of Internet access Finding credible sources Understandability Time constraints Cultural appropriateness	4
Mitra et al. [82]	United States	N = 25	Physical impairment	WWD	Individual semi-structured interview	To examine unmet healthcare needs during and around the time of pregnancy among a sample of women with physical disabilities	Three themes: 1. Clinician knowledge and attitudes 2. Physical accessibility of health care facilities and equipment 3. Need for information related to pregnancy and postpartum supports	Barriers: Lack of information within the gynecologist community, being viewed as asexual, incapable of bearing children, and being a mother, unwillingness to help WWD, inaccessible medical offices and equipment, having difficulty being in the wheelchair, inaccessible hospital rooms, receiving very little information about prenatal care, postpartum supports and interaction of disability and pregnancy	4
Peters and Cotton [50]	Australia	N = 12	Physical impairment	WWD	In-depth interviews and telephone interviews	To explore barriers in breast cancer screening for women with physical disability	Four themes: 1. Environmental barriers 2. Structural barriers 3. Process barriers 4. System change	Barriers: Difficulties with access to breast cancer screening facilities, lack of lifts and ramps, the inflexibility of the machine, problems with dignity and respect, inadequate education and training	4
Tarasoff [48]	Canada	N = 13 WWD	Physical impairment	WWD	In-depth interviews	To identify the perinatal care experiences and outcomes of women with physical disabilities in one Canadian province	Five themes: 1. Inaccessible care settings 2. Negative attitudes 3. Lack of knowledge and experience 4. Lack of communication and collaboration among providers 5. Misunderstandings of disability and disability-related needs	Barriers: Difficulty with finding information, lack of communication and collaboration among providers, inability to be pregnant , inaccessible assistive devices, inaccessible perinatal care settings, feelings of anxiety during pregnancy, lack of knowledge and experience, lack of understanding of disability and disability related needs, being uninterested in consulting	3
Mitra et al. [39]	United States	N = 14	Physical impairment	Healthcare Providers	semi-structured telephone interviews	To explore the maternity care experiences of women with physical disabilities	Four themes: 1. Practitioner level clinical 2. Practice level system level 3. Lack of scientific evidence	Barriers: Unwillingness to provide care Problems with office equipment like adjustable exam tables Time limits, Insurance reimbursement policies Lack of disability specific clinical information and data on the interaction of disability and pregnancy, lack of maternity practice guides, lack of training, and education	5
Dean et al. [54]	India	N = 22	Physical impairment	WWD	individual In-depth interviews	To explore commonality and heterogeneity in the experiences of disabled women in relation to their sexual and reproductive needs and rights	Two themes: 1. Decision making 2. Sexual and reproductive health service use	Barriers: Awareness and perceived need, autonomy in decision making, fear of poor treatment in state-run facilities, the derogatory language of medical staff, lack of money to pay for private care, negative behaviors, Physical accessibility challenges,	3
Methley et al. [56]	UK	N = 24 (F: 19 , M: 5) People with MS N = 34 (F: 28 , M: 6) Healthcare professionals	Physical impairment	WWD Healthcare providers	individual Semi-structured interviews	To explore perspectives and experiences of people with MS and healthcare professionals of UK healthcare services for MS	Three themes: 1. Access 2. Interpersonal interactions 3. Continuity of care	Barriers: Identification of candidacy, navigation, permeability, adjudications, appearances at health care services, Offers and resistance, operating conditions (Local structural and financial influences on service provision)	5
Malouf et al. [32]	UK	N = 9	Intellectual impairment	WWD	In-depth semi-structured interviews	To explore the lived experiences of pregnancy, childbirth, prenatal and postnatal care and services received by this women with learning disability	Four themes: 1. I hate being treated differently 2. I find it harder to understand than other people 3. We’ve had to prove ourselves 4. Make sure you’ve got very good support around you	Barriers: Disrespectful attitudes and denial of choice, problem with understanding of normal care, the lack of needed written information, problem with verbal communication, negative reactions from the members of the family, and Being judged by professionals, discrimination to safeguard their children	4
Schildberger et al. [40]	Austria	N = 10	Physical and sensory impairment	WWD	In-depth individual, semi-structured interviews	To examine the experiences of women with mobility or sensory impairments with regard to pregnancy, childbirth and the puerperium in Austria	Three themes: 1. Social networks 2. Self-efficacy and self-awareness 3. Communication, transparency and information	Barriers: discriminatory attitudes, lack of support and lack of confidence, Anxiety, uncertainty and awkwardness, lack of verbal or non-verbal communication, inadequate information about per partum care, lack of knowledge	5
Soltani et al. [41]	Iran	N = 50	Physical and Intellectual impairment	WWD Healthcare providers Policy makers	individual Semi-structured interviews	To identify cultural barriers in access to healthcare services for people with disability in Iran	Three themes: 1. Personal barriers 2. Structural barriers 3. Social barriers	Barriers: Providers’ Reluctance to provide health services, to humiliate PWD when receiving health care, Misconceptions about disability, discrimination towards disability	3
Gartrell et al. [30]	Cambodia	N = 33	Physical, sensory and Intellectual impairment	WWD	In-depth interviews and Focus group interviews (N = 1)	To provide foundational understanding of persons with disabilities’ current sexual and reproductive health care and needs and to inform future project Interventions	One theme: 1. Access	Barriers: Lack of physical, communicative and financial access, low access to information	3

Seven studies were set in North America, seven in Europe, five in Asia, two in Africa, and two in Australia. Twenty one were conducted in an urban setting and two in a rural setting. Eight studies were conducted to identify barriers in access to maternal care, six in access to breast cancer screening services, three in sexual and reproductive health services and six in other general healthcare facilities. In the 24 included studies, a total of 492 WWD were included in the overall sample. The categorization of main findings of the literature has been shown in Table 3.

**Table 3:** Categorization of main findings of the included studies

Dimensions	Personal barriers	Structural barriers
Approachability	Difficulty to use available information Limited knowledge	Lack of the needed Information Lack of Transparency Using unfamiliar biomedical jargon Limited Knowledge Lack of experience
Acceptability	Lack of autonomy Distrust Physical discomfort Social isolation Cognitive deficits Past negative experiences Stress and anxiety Embarrassment Feeling of pain and being tortured	Insufficient social supports Erroneous assumptions Negative attitudes Stigma Discriminatory attitudes Being judge Being ignored Reluctance to provide care Violence or abuse Verbal, physical and sexual abuse Impoliteness/rudeness Insult
Availability	Not applicable	Inaccessible equipment Transportation Lack of Internet access Physical access Lack of maternity practice guides Lack of assistive devices in healthcare settings Lack of consultation and/or notification
Affordability	Unaffordability to pay for private healthcare Poverty Financial dependence High transportation costs Being single	Insurance reimbursement Lack of insurance coverage
Appropriateness	Communicative problems Low health literacy	Disconnected services Lack of communicative tools in healthcare settings Lack of skills and trainings among providers

### Approachability

In this dimension, four factors of poor knowledge, negative experiences, limited information and lack of transparency limited access to health services for WWD. Women’s limited knowledge and their cognitive, hearing or visual impairments intensified their problems to utilize healthcare.

In both developed and developing countries, WWD reported different problems in accessing health information [30, 32, 39–41]. In developing countries, like Cambodia, WWD who lived in the rural areas reported different patterns in access to services like sexual and reproductive health information. The main source of information was their social network of families, neighbors and friends. For example, to learn about menstruation, WWD would listen to the conversation of older mothers [30]^.^ In such countries, non-governmental organizations (NGOs) had a secondary role in providing information about maternal care for WWD.

In some studies, mothers were able to gain information on the internet or through their friends and family members. In the study by Malouf et al., women with intellectual disabilities were given easy to read information. Some of them could text their midwife with any questions and some would participate in antenatal and postnatal classes to obtain needed information [32]. In some studies, WWD mentioned that healthcare staff did not provide adequate explanation about the procedures like signing a consent form [32, 42]. Remembering the details of the appointments and conversations with healthcare providers was a considerable problem for women with cognitive impairments. This problem would lead to insufficient maternity care utilization and missed appointments [43]. Also, the findings of Lee et al. [44] showed that healthcare professionals found it difficult to transfer information to understand the needs of people who have hearing loss or intellectual disabilities.

Knowledge was a remarkable barrier in access to healthcare for WWD. Many studies indicated that many service providers lack the capacity to understand and fulfill the needs of WWD regarding their sexual reproductive health (SRH) and their breast cancer screening services [40, 42–46]. In the study by Ganle et al. [47] in Ghana, physicians noted that they are well informed and up to date on chronic diseases, such as diabetes and hypertension, but they do not see a lot of patients with disabilities.

Also, some studies indicate that women with intellectual disabilities had a limited knowledge of the healthcare providers and the needed care like SRH and breast mammography. Their awareness of health issues such as preventive and risk factors, signs and symptoms were limited to a few sources of information including nursing staff and their friends. The socioeconomic status and the kind and severity of their disability had a key role in women’s knowledge [30, 32].

### Acceptability

In this dimension, various factors such as insufficient social supports, erroneous assumptions, being ignored, discriminatory attitudes, lack of choices and preferences, confidence, stigma, violence or abuse, social isolation, negative past experiences, anxiety and embarrassment, and cognitive deficits limited access to health services for WWD .

Many studies showed that there are erroneous assumptions and attitudes existed toward PWD [39, 40, 44, 48]. Some findings in this review showed that service providers believe that women with intellectual disabilities or/and visually impaired people were not able to be pregnant, to look after a baby, to perform safe sexual activities, to make a decision and to give birth naturally [40, 48].

Abuse in both healthcare and family settings was one of the most important obstacles in access to healthcare among WWD [30, 43, 49]. The findings of Bradbury et al. indicate that women with learning disability face violence and domestic abuses [43]. Participants noted that they experience different kinds of emotional, psychological, and physical violence. Some WWD, because of their cognitive disabilities, would not understand the nature of domestic abuse. Also, domestic abuse would affect the quality of their interpersonal relationships while also creating fear, stigma and misconception during the provision of health services [49]. It is important to note that, violence is not limited to domestic abuses.

Studies indicated that the women with intellectual disabilities faced barriers in making informed decisions. Health providers sometimes ignored their preferences to choose needed healthcare. Some WWD are not given the enough time and information to have choice and they feel under pressure to make decisions. Also, Megasi and Hummel found that, some families would try to control the decisions and lifestyles of WWD, which in turn, resulted in a loss of motivation, volition and independence among WWD [31].

Furthermore, the studies found that social isolation, coupled with living with a disability, may lead to a form of social oppression, which in turn hampers access to healthcare for WWD [31, 50]. The findings of Neille and Penn in South Africa showed that different factors such as inability to make and develop intimate relationships, loss of friendships, exclusion from family activities and feelings of isolation could lead to social exclusion [51].

In addition to socio-cultural problems mentioned above, studies indicated that stigma was a major factor to poorer access to healthcare for WWD. Allen et al. [52] revealed that the women’s feeling of stigma was related to different factors like poverty, being uninsured, inability to buy a health insurance on their own (or kind of coverage), receiving public assistance, an internal sense of inefficiency, and health providers’ disrespectful interactions with WWD.

### Availability

This dimension explored whether accommodations are available and whether or not health services are available in the right place and at the time that they are needed. In this dimension the factors such as inaccessible equipment, lack of physical access to transportation systems and buildings, lack of internet access, lack of maternity practice guides, lack of assistive devices in healthcare settings and lack of consultation and/or notification impacted healthcare access for WWD.

One of the important barriers in this dimension was related to scientific evidence. Many studies highlight that there is a general lack of existing evidence and knowledge on maternal care for WWD. Mitra et al. [39] found that lack of clinical guidelines and disability-specific clinical data and information on issues like pregnancy in women with physical disabilities are serious challenges for providers.

Transportation, especially in developing countries, was mentioned as one the most important barriers to physical access to healthcare facilities. Peters and Cotton [50] described transportation as an important facilitator to improve access to breast screening facilities. Access to transportation would influence the women’s decisions to return for screenings. The long travel distances prevent WWD to accessing healthcare facilities in urban areas [51]. Also the findings of Lee et al. in the Philippines showed that the WWD report more dependence to their family members for movement and transportation to SRH services than their counterparts without disabilities.

Additionally, Coffey et al. noted that some participants encounter a lack of internet access to health information. Finding credible sources, available time, language and the cultural appropriateness of information were mentioned as the most common obstacles of access to information sources [53].

### Affordability

In this dimension, factors such as poverty, unemployment, financial dependence, being single, high transportation costs, and lack of insurance coverage were identified as the main barriers of access to healthcare for WWD. Additionally, negative cultural issues, especially in the developing countries, would intensify this problem so that some people would steal the WWD’s belongings because they were deemed alone, weak and disabled.

Financial problems such as poverty, financial dependence and high cost services were identified. In some studies conducted in Asian countries, like Cambodia, poverty was cited as a remarkable factor to use SRH. Findings of this study showed that women who were single, did not have any children and social support, were more likely to report poorer access compared to others. Cultural factors had a considerable role in financial problems of WWD. For example in Gartrell’s [30] study, one of the WWD who was single and had neither parents nor older siblings noted that her neighbors used to steal her jewelry.

The review of the studies indicate that financial dependence may be a major barrier to utilize healthcare services. WWD usually are unemployed and are not able to pay for needed services. In addition, they belong to low income families in which their household members are unemployed or earn income in informal sectors [30, 41]. The findings of Dean et al. ,in India, showed that WWD with lower socio-economic status have to receive their SRH services in government facilities that provide poorer quality care than private sector facilities [54].

### Appropriateness

WWD, due to cognitive, hearing and visual impairments were not able to communicate with health professionals effectively. But factors like low health literacy, lack of communicative tools in healthcare settings and lack of necessary skills and trainings among health providers to communicate with WWD were identified as the significant barriers in access to healthcare for WWD.

In this review, we identified factors that could limit access to healthcare for WWD [32, 40, 43]^.^ Communication problems, like unfamiliar biomedical jargon and lack of health literacy were two important factors cited frequently in the studies. In the study by Barr et al., discomfort about communication issues was reported by many of WWD, except those with cognitive disabilities who lived in the group homes [55]. Lack of sensitivity among healthcare staff in the mammography process, like being touched by staff, positioning and undressing would cause stress, anxiety and fear during mammography for WWD.

Some studies highlighted the personal aspects of communication problems [45, 51, 56, 57]. For example, Mcilfataric et al. [42] found that women’s cognitive deficits and level of their understanding were obstacles to accessing breast screening services. In other studies, there were different experiences of interactions with healthcare staff. In many cases, the negative interactions occurred due to poor interpersonal skills of healthcare staff like general practitioners and nurses. Reluctance, humiliation, insult, violence, physical abuse, lack of respect, empathy and politeness were among the cases cited by WWD in the different studies [39, 56].

Also, the findings show that interpersonal relationships are affected by the lack of appropriate communication tools. According to type of disability, the needs of WWD were different. For example Bradbury-Jones et al. found that speaking to some participants with communication impairment is more difficult than others. Thus some WWD needed written and pictorial information to seek their services and some needed hearing aids [49]. Consequently, communication challenges for WWD would cause them to bring a family member to provide communication supports. Furthermore, using medical expression and unknown jargon by healthcare professionals made it difficult to access healthcare for to women, in particular those with learning disabilities [49].

## Discussion

The aim of this study was to identify barriers in access to healthcare for WWD through the systematic review of qualitative research. In this study, we intended to make a complete and clear picture of the most important barriers in access to healthcare for WWD internationally from qualitative research findings. The findings of the reviewed studies demonstrate that WWD need a variety of supports to better access to healthcare. In this review WWD reported different problems to utilizing breast cancer screening, SRH services, rehabilitation services and maternal care.

WWID, because of cognitive deficits, experienced low health literacy and significant communication problems to access services like mammography or SRH services [30, 42, 58]. Communication issues caused problems with seeking the needed information and health services. Communication problems not only would reduce effective interaction between a WWD and their health providers, it also would reduce their likelihood of going to healthcare facilities [39, 42, 44, 55, 57].

We found that WWD as consumers, providers and health systems form three main dimensions of the communication challenges. Personal factors like cognitive, mobility and sensory impairments limit women’s ability to seeking and understanding the needed information [42, 45]. Lack of awareness and knowledge among healthcare providers about disability and the proper methods of communication with WWD would affect the quantity and quality of interpersonal relationships between providers and WWD [42, 46, 49]. Our healthcare systems should develop their capacity to facilitate interpersonal relationship through providing substructures, education courses and various communication tools so that all people with different disabilities could have a satisfactory and effective relationship with their providers.

Some studies in this review indicated that socio-cultural factors could have a major role in poor access to healthcare for WWD [11, 30, 32, 44, 57]. Maternal status and age in low income countries like Cambodia affected access to health services so that single, young women had limited knowledge about SRH services and felt embarrassed when speaking about their SRH problems [30].

WWD living in rural areas face deeper problems to receive the needed information and services like breast cancer screening and SRH services. WWD and their families needed an adaptable and affordable transportation system to move safely from their homes to the healthcare facilities. Some studies reported that some healthcare services including rehabilitation, SRH and mammography services were not sufficient for WWD. In many countries like Pakistan, Cambodia, India, Ghana, Philippine and Nepal, these services usually are provided in the central parts of cities and WWD have to travel a long distance to use the needed services [11, 30, 44, 45, 54, 57, 59]. Also, WWD identified environmental barriers, lack of adaptable equipment, and insufficient allocation of time in the studies. Some studies noted that WWD had a low level of autonomy to choose their providers and services. Often, a member of family accompanies WWD when traveling and receiving healthcare [31, 32, 43, 49, 54].

The studies show that women with cognitive, vision and hearing impairments face special barriers to access to healthcare facilities. Governments and health systems should have specific policies to accommodate for all forms of disabilities. Healthcare services need to be accessible for disadvantaged groups in society. WWD, like women without disabilities, have similar rights to be a parent, to have a child, to look after their babies on their own. In some studies, WWD had to prove their family members and the authorities that they have the needed qualifications to be a suitable parent [32, 45, 60]. For this, advocacy from the PWD, families, NGOs, and public organizations is necessary to support the rights of WWD.

Many studies cited that WWD faced financial problems when accessing healthcare. In some studies, WWD especially those who were married, usually relied on their family income and reported better access to different financial resources in comparison to single women with disabilities. Often, WWD were unemployed and did not have any income. Many WWD were especially worried about the future, the cost of healthcare services and financial uncertainty in their old age [30, 32, 45, 57, 60]. In some studies, WWD reported that they had to spend more on transportation because they were unable to use public transportation such as buses and trains [41, 61]. Furthermore, WWD faced large out of pocket payments for services like rehabilitation and dental care because there was no coverage for them [41, 61]. Also, some WWD had difficulty in proving their financial eligibility to gain financial assistances.

It is notable that, various quantitative studies have been done about extra costs of living with disability. Some of the studies note that older adults with disabilities face higher out of pocket payments and transportation costs in comparison to other age groups [62, 63]. Mitra et al. [64] revealed that the estimated extra costs of disability as a percentage of mean annual income vary from 12% in Vietnam to 40% for older adult households in Ireland. In another study, Morris and Zaidi estimated the extra costs of disability in European countries around 44 and less than 30% of income for a household with an adult reporting a work-related disability and a household with an adult who receives disability benefits respectively [65].

This review of the qualitative literature identified barriers to healthcare access for WWD related to personal factors, as well as great limitations in the capacity of healthcare providers and healthcare systems to adequately provide care for all consumers, including WWD. In order to impact these great disparities, there is a need for healthcare systems and larger society to recognize the social model of disability [66]. The social model of disability aligns with the World Health Organizations International Classification of Functioning, Disability, and Health (ICF), in acknowledging that limitations in participation for PWD is largely defined by the environment and not their disability itself [67]. Approaching the design and delivery of care utilizing concepts from Universal Design [68], would not only ensure care was accessible for WWD, but for all healthcare consumers whom providers may or may not struggle with health literacy skills.

It must be noted that women without disabilities experience some similar challenges to use healthcare in comparison to WWD. In general, some variables such as age (being older), socioeconomic factors (low income and low payment), marital status, household dimension, education (being illiterate) and employment status (job insecurity and job instability) affect access to healthcare for women without disabilities as well [69–76]. Financial dependence and economic factors are considered as one of the most significant factors in access to health services for women with and without disabilities [70, 71, 75]. Women are more likely than men to be uninsured and unemployed [69, 77, 78]. In total, gender and the role of gender in access to healthcare have been discussed in the different studies [69]. We should note that women with and without disabilities compared to men have different problems and different patterns of needs and illness that must be considered in the health policy processes.

## Limitations

In this systematic review, we faced some problems to investigate and interpret the findings of included studies. First, in some studies, demographic characteristics of participants like age, severity of disability, marital and maternal status, household’s characteristics, education and occupational status had not been provided precisely. Thus we found it difficult to fully discuss the facilitators and obstacles affecting access to healthcare for WWD. Second, because of the qualitative nature of the included studies, we were not able to report any related quantitative estimates. Third, some studies have not provided the clear categorization of their findings making it difficult to identify and report their themes and subthemes. Fourth, the studies had been conducted in the different socio–economic contexts thus we were not able to generalize the mentioned barriers in a study to the other studies. Additionally, this study focused specifically on barriers to healthcare for WWD, future studies and reviews can include discussion of facilitators to healthcare for WWD. Also, we suggest more studies to investigate barriers to access to medications and other healthcare services among different groups of disabilities.

## Conclusion

The findings show that WWD not only experience financial and physical barriers in access to healthcare, but also they face discriminatory and disrespectful behaviors from health professionals. Healthcare systems need to have respect for the inherent dignity of WWD, pay attention to their preferences and choices, provide non –discriminatory and respectful treatment, work on attitudinal changes and update the training of health care staff for working with WWD. Families and communities also should participate in the advocacy efforts supporting WWD in their desired access to health care.

## Data Availability

The datasets generated and/or analyzed during the current study are not publicly available but are available from the corresponding author on reasonable request.

## Abbreviations

ICF: International Classification of Functioning, Disability, and Health
WWD: Women with Disabilities
PWD: People with Disabilities
WWID: Women with Intellectual Disabilities
PWID: People with Intellectual Disabilities
SRH: Sexual and Reproductive Health
NGOs: Non-Governmental Organizations
COREQ: Consolidated Criteria for reporting Qualitative Research
